# 

**Published:** 1913-12-20

**Authors:** 


### CONTENTS.

PAGE

**King Edward's Hospital Fund** ... ... ... 299

**Censorship to an Extreme** ... ... ... ... 300

**Hospital and Institutional News** ... ... ... 301

The Queen at Derby Infirmary—St. George's Hospital—"Truth" Doll Exhibition—A Pension Fund for Hospital Officers—"Starved to Death" in a Sanatorium—A Windfall for the London Hospital—The Liverpool Tuberculosis Scheme—A Clearing Hospital Complete—The Surrey County Hospital's Jubilee—Modern Hospital Musical Societies—Out-Patient Restaurants—L.C.C. and Nursing Homes: Parliamentary Bill—London's Latest Pay Hospital—Southwark Guardians and Guy's Hospital: A Suggestion—Central Association for the Mentally Defective—Hampstead's New Health Institute—Alternatives to the Tenement System—Town Trees and Public Health—The Order of St. John—Hospital for Babies—The Clapham Maternity Hospital—Hospital Officers' Conflicting Opinions—The Insurance of Radium—Our Prizes and the Special Appeal Number—This Week's Drug Market.

**Hospital Prophylaxis: The Prevention of Accidents** ... ... ... ... ... ... 307

PAGE

**The Mortality from Phthisis** ... ... ... 308

The Question of Personal Infection.

**Out-Patient Statistics and Cost** ... .. ... 309

Supplementary Memorandum issued by the Hospital Funds.

**The County Council and an Ambulance Service** 310

The Report and Debate at Tuesday's Meeting.

**Report on the Lowestoft Hospital** ... ... 311

By Sir HENRY BURDETT, K.C.B., K.C.V.O.

**National Insurance Act Developments** ... ... 313

Deficiencies and How They Will be Made Good.

**King Edward's Hospital Fund for London: Award of Grants** ... ... ... ... ... 315

The Wernher Windfall.
The League of Mercy's Report.
The Distribution Committee's Report.
Grants Recommended by the Distribution Committee.
The Convalescent Homes Committee's Report.
Grants Recommended by the Convalescent Homes Committee.
The Duke of Teck's Speech.

### CONTENTS—(*continued*).

| | PAGE |
|---|---|
| **Obituary** ... ... ... ... ... ... ... ... | 320 |
| **The Editor's Letter-Box** ... ... ... ... ... | 321 |
| Discontent in Mental Hospitals. | |
| Dr. Walsh's Proposed Laboratory Clinic. | |
| **The New Empire Pay Hospital** ... ... ... ... | 321 |
| **"Harley Institute"** ... ... ... ... ... ... | 321 |
| **Staff Changes at Camberwell Infirmary** ... | 321 |
| **A Winter in the Upper Engadine, 1885** ... | 322 |
| **Appointments** ... ... ... ... ... ... ... | 323 |
| **Hospital Almoners' Council** ... ... ... ... | 323 |
| **Gifts and Bequests** ... ... ... ... ... ... | 323 |
| **A Successful Matinee** ... ... ... ... ... | 323 |
| **Ancoats Hospital and Amateur Actors** ... | 323 |
| **The Profession of Medicine** ... ... ... ... | 324 |
| The Approved Societies and their Medical Referees. | |
| The National Medical Union: Meeting of the Provisional Council. | |
| Trade or a Learned Profession? | |
| **The Institutional Worker** ... ... (Suppl.) | 1 |
| A New Laundry Disinfectant and Bleach. | |
| Apparatus for Handling Radium. | |
| **Our Bureau of Information** ... ... ,, | 2 |
| Questions for December. | |
| Accelerated Hot-Water System. | |
| The Sick and in Need. | |
| Employment and Training. | |
| **Buildings Contemplated and in Progress** ,, | 3 |
| **The Book Market** ... ... ... ... ,, | 3 |

